# Rehabilitation Services for Young-Onset Dementia: Examples from High- and Low–Middle-Income Countries

**DOI:** 10.3390/ijerph21060790

**Published:** 2024-06-17

**Authors:** Aida Suárez-González, Sharon A Savage, Suvarna Alladi, Viviane Amaral-Carvalho, Faheem Arshad, Julieta Camino, Paulo Caramelli, Adelina Comas-Herrera, Julia Cook, Claudia Cooper, Laura García Díaz, Stephanie M. Grasso, Regina Jokel, Monica Lavoie, Tomás León, Thomas Priya, Teresita Ramos Franco, Cathleen Taylor-Rubin, Rosemary Townsend, Angelika Thöne-Otto, Andrea Slachevsky, Anna Volkmer, Wendy Weidner, Claire MC O’Connor

**Affiliations:** 1 Dementia Research Centre, UCL Queen Square Institute of Neurology, University College London (UCL), London WC1N 3BG, UK; 2 School of Psychological Sciences, College of Engineering, Science and Environment, The University of Newcastle, Callaghan, NSW 2308, Australia; sharon.savage@newcastle.edu.au; 3 Department of Neurology, National Institute of Mental Health and Neurosciences (NIMHANS), Hosur Road, Bangalore 560030, India; 4 Departamento de Neurologia, Faculdade de Medicina da Universidade de São Paulo, São Paulo 01246-903, SP, Brazil; 5 Behavioral and Cognitive Neurology Unit, Faculdade de Medicina, Universidade Federal de Minas Gerais, Belo Horizonte 30130-100, MG, Brazil; 6 Institute of Health and Social Care, London South Bank University, London SE1 0AA, UK; 7 School of Health Sciences, Faculty of Medicine and Health Sciences, University of East Anglia, Norwich NR4 7TQ, UK; 8 Care Policy and Evaluation Centre, London School of Economics and Political Science, London WC2A 2AZ, UK; 9 Herefordshire and Worcestershire Health and Care NHS Trust, Worcester WR5 1JR, UK; 10 Centre for Psychiatry and Mental Health, Wolfson Institute of Population Health, Queen Mary University of London, London EC1M 6BQ, UK; 11 School of Rehabilitation Science, McMaster University, Hamilton, ON L8S 1C7, Canada; 12 Department of Speech, Language and Hearing Sciences, University of Texas, Austin, TX 78712-1069, USA; 13 Rotman Research Institute, Toronto, ON M6A 2X8, Canada; 14 Faculty of Medicine, University of Toronto, Toronto, ON M5S 1A8, Canada; 15 Baycrest Health Sciences, Toronto, ON M6A 2E1, Canada; 16 Chaire de Recherche sur les Aphasies Primaires Progressives—Fondation de la Famille LEMAIRE, CHU de Québec-Université Laval, Québec, QC G1V 0A6, Canada; monica.lavoie.1@ulaval.ca; 17 Memory Unit, Neurology Department, Memory and Neuropsychiatric Center (CMYN), Hospital del Salvador and Faculty of Medicine, University of Chile, Santiago 7500922, Chile; 18 Department of Psychiatry and Global Brain Health Institute, Trinity College, D02 K104 Dublin, Ireland; 19 Department of Psychiatric Social Work, National Institute of Mental Health and Neurosciences (NIMHANS), Hosur Road, Bangalore 560029, India; priyathomasat@gmail.com; 20 Speech Pathology Department, War Memorial Hospital, Sydney, NSW 2024, Australia; 21 School of Psychological Sciences, Macquarie University, Sydney, NSW 2109, Australia; 22 Dyscover Ltd., Leatherhead KT22 0BN, UK; 23 Clinic for Cognitive Neurology, University Hospital Leipzig, 04103 Leipzig, Germany; 24 Max-Planck Institute for Human Cognitive and Brain Sciences, 04103 Leipzig, Germany; 25 Geroscience Center for Brain Health and Metabolism (GERO), Santiago 7500922, Chile; 26 Neuropsychology and Clinical Neuroscience Laboratory (LANNEC), Physiopatology Program—Institute of Biomedical Sciences (ICBM), Neuroscience and East Neuroscience Departments, Faculty of Medicine, University of Chile, Santiago 7500922, Chile; 27 Neurology and Psychiatry Department, Clínica Alemana-Universidad Desarrollo, Santiago 7650568, Chile; 28 Division of Psychology and Language Sciences, University College London (UCL), London WC1H 0AP, UK; 29 Alzheimer’s Disease International, London SE1 4PU, UK; 30 Centre for Positive Ageing, HammondCare, Sydney, NSW 2170, Australia; 31 School of Psychology, Faculty of Sciences, University of New South Wales, Sydney, NSW 2052, Australia; 32 Neuroscience Research Australia, Sydney, NSW 2031, Australia

**Keywords:** young-onset dementia, rehabilitation, Alzheimer, frontotemporal dementia, primary progressive aphasia, posterior cortical atrophy

## Abstract

The WHO Dementia Global Action Plan states that rehabilitation services for dementia are required to promote health, reduce disability, and maintain quality of life for those living with dementia. Current services, however, are scarce, particularly for people with young-onset dementia (YOD). This article, written by an international group of multidisciplinary dementia specialists, offers a three-part overview to promote the development of rehabilitation services for YOD. Firstly, we provide a synthesis of knowledge on current evidence-based rehabilitative therapies for early-onset Alzheimer’s disease (EOAD), behavioural variant frontotemporal dementia (bvFTD), primary progressive aphasia (PPA), and posterior cortical atrophy (PCA). Secondly, we discuss the characteristics of rehabilitation services for YOD, providing examples across three continents for how these services can be embedded in existing settings and the different roles of the rehabilitation multidisciplinary team. Lastly, we conclude by highlighting the potential of telehealth in making rehabilitation services more accessible for people with YOD. Overall, with this paper, we aim to encourage clinical leads to begin introducing at least some rehabilitation into their services, leveraging existing resources and finding support in the collective expertise of the broader multidisciplinary dementia professional community.

## 1. Introduction

Rehabilitation refers to a set of interventions designed to optimise functioning and reduce disability in individuals with health conditions [1]. The World Brain Day 2023 campaign, under the auspices of the World Federation of Neurology and the World Federation of Neurorehabilitation, stressed the need for rehabilitation for people living with neurological conditions, including dementia [2]. Although rehabilitation cannot stop the progression of dementia, its importance in improving functional abilities and promoting health and quality of life has gained international recognition in recent years. This recognition is supported by high-quality randomised clinical trials proving its effectiveness [3,4,5,6]. Indeed, a package of interventions for rehabilitation in dementia has recently been released by the WHO [7] outlining the required resources to deliver them. Rehabilitation in dementia, also called restorative care or reablement [8], involves (1) personalised and meaningful goal setting to ensure that each individual’s programme is tailored to their preferences and abilities [9,10,11], (2) a set of enhanced learning, behavioural, and environmental modification and compensatory techniques aimed at reducing disability, and (3) working in partnership with the person living with dementia and their family [5].

Young-onset dementia (YOD) is any form of dementia that clinically manifests before the age of 65 [12]. The current global prevalence is 119/100,000 inhabitants [13]. The earlier timing of symptom onset brings different social, financial, and occupational challenges compared to later-onset dementia presentations, since people with YOD are often still reliant on full-time employment income and have caring responsibilities [14,15]. The most common form of YOD is the typical amnestic presentation of early-onset Alzheimer’s disease (EOAD), characterised by progressive impairment of episodic memory that impacts the ability to acquire new information, recall recent events, and orient oneself in time and place [16,17]. Behavioural variant frontotemporal dementia (bvFTD) is the most common subtype of FTD, wherein insidious changes arise in personality and behaviour [18], followed by the primary progressive aphasias (PPA) [19], involving a decline in speech and language abilities. Three PPA phenotypes have been described to date, characterised by semantic breakdown (semantic variant PPA, svPPA), non-fluent speech with agrammatism (non-fluent/agrammatic variant PPA, nfvPPA), and impaired repetition with phonologic errors (logopenic variant PPA, lvPPA) [19]. Posterior cortical atrophy (PCA), also called the visual variant of AD, is a less frequent form of YOD. PCA causes prominent and progressive visuospatial and perceptual dysfunction and impairment of other functions associated with parieto-occipital cortices [20,21]. 

Within this paper, we review interventions aimed at promoting health through the reduction of cognitive disability associated with these YOD phenotypes, rather than interventions aimed at addressing issues of mood or adjustment to diagnosis. We also discuss how these interventions can be delivered in different settings and contexts, including how to leverage telehealth to increase access to therapy. 

## 2. Evidence-Based Therapies

In the sections below, we outline current rehabilitation therapies for each YOD phenotype.

### 2.1. Amnestic Early-Onset Alzheimer’s Disease

As in later-onset amnestic AD, people with typical amnestic EOAD show the greatest difficulties with episodic memory, accompanied by a less prominent decline across other cognitive domains [22]. To date, few studies have specifically focused on rehabilitation in EOAD, with evidence limited to a small number of single cases, small cohort studies, and mixed-YOD group studies where participants with EOAD are predominant [23,24,25]. However, people with EOAD have been included as part of large clinical trials showing significant and lasting benefits from cognitive rehabilitation [26,27]. Most of these studies have involved people with mild to moderate stages of AD.

People with amnestic EOAD may benefit from cognitive rehabilitation to address specific identified goals within their everyday functioning [26,27,28]. For example, learning how to orient oneself in time or place, organising daily functional activities, remembering the location of important items, or learning how to use a new assistive device. Cognitive rehabilitation in EOAD is delivered using techniques such as modelling, action-based learning, and graded activity [27,29]. 

Once learned, assistive technology such as digital calendars, simplified TV remotes, mobile phones, and stove timers can help people with EOAD to orient and navigate around the neighbourhood or local shops [30], complete shopping tasks (e.g., form grocery lists to assist memory for purchasing items) [31], improve safety within the household, and provide reminders to complete everyday tasks [32,33]. Vocational rehabilitation may also be considered, with some evidence indicating safe, supported engagement in workplace contexts such as retail [34,35].

### 2.2. Behavioural Variant Frontotemporal Dementia

People with bvFTD experience functional decline across basic and complex activities of daily living, in parallel with their behaviour and cognitive changes [36]. To date, behaviour management and carer interventions have been the primary focus within the nonpharmacological management of bvFTD [37,38,39]. However, rehabilitation interventions addressing both functional and behavioural symptoms in people with bvFTD are showing promising results [40,41,42,43]. In particular, the occupational therapy-based intervention, Tailored Activity Program (TAP) [44], may help maintain everyday functioning and reduce behaviour symptoms in FTD [42,45]. TAP involves working collaboratively with family carers to prescribe personalised activities for behaviour management. Behaviour modification strategies can be developed based on contingency management, where a person with bvFTD is rewarded with something they value after they have completed a certain activity of daily living (see a full description with examples in O’Connor et al. 2016 [45]). When setting up interventions for bvFTD, understanding each individual’s cognitive and behavioural profile (e.g., apathy subtype) is pivotal to effective personalised care [46,47].

People with bvFTD may also benefit from Positive Behaviour Support (PBS) therapy [48,49]. PBS involves a functional behaviour assessment to identify factors underlying behaviours that are difficult to manage. This assessment informs a multicomponent PBS plan involving a review of the person–environment fit, teaching positive skills that are functionally related to the target behaviour, considering antecedents, promoting desired behaviour, and providing strategies for how to react to the behaviour when it occurs [49]. 

### 2.3. Primary Progressive Aphasia 

People with PPA experience a range of speech and language changes that impact social relationships and engagement [50,51]. While some language deficits, like word retrieval difficulties, may be shared among the three PPA phenotypes, other aspects are phenotype-specific (e.g., non-fluent, halting speech, and poverty of expression in the nfvPPA; single-word comprehension deficits within the svPPA), such that distinct rehabilitation approaches are required. In addition, across the three phenotypes, there are differences in the broader cognitive profile and expected evolution that should be considered when selecting a suitable rehabilitation programme [52,53]. There is a rich body of evidence suggesting significant and long-lasting benefits of behavioural approaches to reduce communication disability and promote quality of life for people with PPA in mild and moderate stages [54,55,56,57,58,59,60,61,62]. Adherence to treatment is higher when treatment starts soon after diagnosis [58].

The most common rehabilitation approaches described in the literature across the different subtypes of PPA are word retrieval therapies, which can be effectively delivered either face to face or via technology-supported methods [54,55,56,63,64,65]. Lexical retrieval techniques involve repetitively completing “look and repeat” training (e.g., presentations of picture–word pairings) or cueing (e.g., phonological, orthographic, or semantic cues). Despite disease progression, gains from treatment can be maintained for up to 12 months [55], with booster sessions required in some cases [60]. Moreover, a prophylactic effect, slowing the decline in intact skills, has been observed from naming therapies [66,67]. 

People with PPA may also benefit from developing and practising scripts (script training) to support anticipated conversations. Script training has been successfully adapted to nfvPPA, with preliminary evidence showing lasting and generalisable gains [63]. In clinical practice, therapists also use compensatory approaches, such as communication partner training (CPT) [68,69] and group-based delivery of therapy. Group-based delivery is a beneficial [70,71,72] and sustainable approach [57,73] that has shown positive results in functional communication, social discourse, and the use of multi-modal communication methods [70,71,72].

### 2.4. Posterior Cortical Atrophy

People with PCA are more severely impaired in everyday skills and self-care tasks compared to those with typical AD of the same age and disease duration. This includes greater difficulty using the telephone, putting a coat on, preparing a hot drink, handling money, and using electrical appliances [74,75]. They also typically experience problems with reading due to visual disorientation (e.g., becoming lost on the page) and visual crowding (e.g., words and lines of text cluttering up) [76,77]. Evidence-based recommendations for the rehabilitation of PCA are scarce [78,79,80,81]. However, in practice, a range of techniques from brain injury rehabilitation can be adapted to treat people with PCA (https://www.raredementiasupport.org/wp-content/uploads/2020/03/Home-Safety-Tips-and-Recommendations-Visual-Dysfunction-in-Dementia.pdf, accessed 8 June 2024). 

As with other neurological conditions that involve visual cortical impairment, people with PCA may benefit from using visual cues [82] combined with tactile cues [82] to enhance object location and navigation. This includes using bright stickers on light switches or thermostat buttons [83], placing bright yellow tape at the edge of stairs to facilitate safety going up and down the stairs, or a red mark at the front door to distinguish it from the neighbour’s door [84]. Visual cues can also help reduce disability associated with dressing apraxia by helping to orient a garment in space, indicating which side is the front or what is the correct sleeve for each arm. Similarly, adaptive equipment used by people with disabilities (e.g., plates designed with a high-sided lip to avoid spilling food, level indicator devices to know when a glass has been filled) may assist people with PCA to accomplish activities of daily life. This type of equipment also includes adaptive clothing (e.g., using elastics and Velcro to replace zips, laces, buttons, or sleeveless ponchos to reduce the need for garment orientation) to reduce barriers to dressing [85]. 

The ReadClear app, available in app stores, is a digital aid specifically developed to assist with reading for people with PCA [81]. A clinical trial showed that ReadClear can improve reading for people with PCA through three main text manipulation techniques: the use of a digital reading ruler, greying out the surrounding text around the ruler line, and using wider interline spacing and a shorter length of lines [81]. 

## 3. Models and Delivery of Rehabilitation Services

Although scarce, rehabilitation services for people with dementia do exist and at times provide interventions specific to people with YOD. In the UK, for example, some rehabilitation services for YOD exist in a variety of formats, including as part of reablement services integrated in the social care system, outpatient community teams, memory services, specialised cognitive disorder services, and services offered by third-sector (e.g., charities, social enterprises) organisations [86,87]. There is inconsistency in the provision of these services and wide heterogeneity in the type of therapeutic activities delivered. For instance, whether services are offered in combination (i.e., multidisciplinary) or as stand-alone options, and whether they are delivered as a group or are individualised [86]. 

YOD rehabilitation may be delivered by psychologists, occupational therapists (OTs), speech and language therapists (SLTs), nurses, social workers, and physiotherapists [7,87,88]. The core features of YOD rehabilitation services are the ability to provide age-appropriate and specialist support, integration within existing services, and continuity of care [25,89]. Below, we provide four case studies of YOD rehabilitation services across three continents, reflecting the diverse and flexible approach that can be taken to promote rehabilitation and health in YOD populations (see Table 1 for a summary).

### 3.1. Case Study 1: A Third-Sector Organisation in the UK

The UK National Health Service (NHS) provides public universal healthcare free at the point of delivery. Ten percent of UK residents are also privately insured. There are approximately 214 memory clinics in England [92]. The National Memory Service Accreditation Programme provides standards to benchmark services [93]. These include having a named lead for people with YOD. In the 2019 NHS England National Memory Service Audit [94], 7% of clients were aged under 65, and just over half of services had a named lead for YOD. Existing alongside the NHS funded services, there are other healthcare providers, including third-sector organisations.

In this case study, we report the specialised PPA rehabilitation service provided by Dyscover, an aphasia charity based in Southeast England accepting national referrals (see Case Study 1, Table 1). Dyscover was established in 1994 by an SLT to provide long-term support for people with aphasia after stroke. In 2015, the Dyscover Director and Lead recognised a lack of long-term specialist support for people living with progressive aphasias and initiated a separate, dedicated service based on Life Participation Approach to Primary Progressive Aphasia principles [95]. 

### 3.2. Case Study 2: A Private and Public Collaborating Service in Brazil

In Brazil, the Unified Health System (SUS) provides universal free outpatient and hospital care. Additionally, people may elect to take out supplementary health insurance if they can afford the monthly premiums. There are also private services, generally available in private offices, clinics, and hospitals. 

In this case study, we report the collaborating service offered by the private Specialized Cognitive Service “SER.COG” and the publicly funded Behavioral and Cognitive Neurology Unit, Hospital das Clínicas and Faculdade de Medicina, Universidade Federal de Minas Gerais (UFMG) (see Case Study 2, Table 1).

The UFMG outpatient clinic was founded in 2003 by behavioural neurologists and offers free care services (once a week), alongside research activities. It also offers formal training for healthcare professionals regarding the management of cognitive impairment and dementia. The private service was started in 2019 by its three clinical leaders (neuropsychologist, OT, SLT) who were at the time researchers at the UFMG unit.

### 3.3. Case Study 3: A Public Hospital in Chile

Chile operates a two-tiered health system, comprising both public and private sectors. The public system provides coverage for over 69% of the population and is organised by territory and by level of complexity, ranging from primary to quaternary care. In 2005, a reform known as Universal Access with Explicit Guarantees (AUGE) was implemented, ensuring access to diagnosis and treatment and the right to receive this within certain timeframes. AUGE provides explicit guarantees for 87 health problems established by law which must be fulfilled for all Chileans [96]. In 2019, Alzheimer’s disease and other forms of dementia, including YOD, were incorporated into AUGE [97].

This case study reports the Memory Unit at Hospital del Salvador, in Santiago de Chile, which is integrated into the Memory and Neuropsychiatry Centre (CMYN), a joint initiative between the public health system and the University of Chile (see Case Study 3, Table 1). The purpose of the CMYN is to care for individuals with dementia of all ages, including YOD, provide postgraduate student training, offer continuous education for professionals, and conduct research on neurocognitive disorders. The Memory Unit started in the context of Chile’s National Dementia Plan, launched by the Ministry of Health in 2017. This plan proposed a model of coordinated care for people with dementia and their caregivers, extending from primary care centres to specialised Memory Units. These Memory Units have been implemented in three hospitals nationwide, with the purpose of providing assessment for people with dementia and their caregivers when their health needs cannot be managed at the primary care level. 

### 3.4. Case Study 4: A Public Hospital in India

The healthcare system in India is characterised by a mix of public and private healthcare services. The public sector, primarily represented by government-funded facilities, comprises a network of primary health centres, community health centres, district hospitals, and specialised referral hospitals. These facilities offer subsidised or free healthcare services to the population, especially in rural and underserved areas. The private healthcare sector is vast and diverse, ranging from small clinics to large corporate hospitals offering advanced medical treatments. Private healthcare providers play a significant role in delivering healthcare services, particularly in urban areas, and cater to a considerable portion of the population, including those who can afford to pay out-of-pocket medical expenses or have medical insurance [98].

In this case study, we present the Cognitive Disorders Clinic at the Neurology Department of the National Institute of Mental Health and Neurosciences, a public hospital in Bengaluru, funded by the central government of India (see Case Study 4, Table 1). The Cognitive Disorders Clinic was set up in 2018 by the lead neurologist and her team. The purpose was to provide multidisciplinary care to patients with dementia of all ages including YOD [91,99].

## 4. Using Telehealth to Extend Services

Telehealth involves the use of communications technology (e.g., videocalls) to provide healthcare at a distance [100] and is increasingly utilised for diagnostic and rehabilitation services in YOD [101,102,103,104,105,106]. Telehealth assists in overcoming barriers of geographical distance, an absence of local specialised services (a frequent limitation given the low prevalence of YOD), and travel arrangements to attend frequent clinical appointments (e.g., carers having to take time off). For people with dementia, telehealth has been shown to be a feasible mode of delivery of cognitive rehabilitation [107], occupational therapy [108], and speech and language therapy [73,109,110,111]. Moreover, remote delivery of cognitive rehabilitation reduces time and the travel costs of staff, making rehabilitation cheaper and more sustainable [112]. The adoption of telehealth requires access to the internet and the ability to use technology. In high-income countries, the internet penetration rate is high (≥90%) and in low- and middle-income countries, it is rapidly growing (44% increase since 2020) [113]. A lack of confidence using technology seems to be a more significant barrier for people with dementia and their families to engage with telehealth rather than access to the internet [114]. However, people with YOD belong to a segment of the population with high levels of digital literacy in general [115], and in the cases where this is not true, IT skills may be taught. 

The following two vignettes illustrate real examples of the use of telehealth in PPA and PCA.

*Vignette 1: PPA Telehealth Rehabilitation Therapy*

Jose, a bilingual Spanish–English science teacher, was diagnosed with logopenic variant PPA. His local speech–language pathologists do not know much about PPA and Jose would like to seek services from a bilingual SLT who speaks Spanish. The nearest specialist lives a three-hour drive away but offers therapy via telehealth. During the initial videocall, Jose and the SLT agree that working on lexical retrieval training to address word-finding difficulties is an important component of the treatment plan, alongside communication partner training (CPT). The SLT has Jose and his family virtually “walk” her through their home and his classroom so she can take photographs of objects that are challenging for Jose to name. During teletherapy sessions, the SLT uses these images via virtual screen sharing to practice lexical retrieval of these personally relevant objects. CPT is also delivered over videocall.

*Vignette 2: PCA Telehealth Rehabilitation Therapy*

Aysha has been living with PCA for two years but has not received rehabilitation because there are no OTs specialised in PCA in her area. Aysha’s neurologist invites her to take part in a clinical trial where occupational therapy is delivered via telehealth by OTs based at a specialised centre overseas. Aysha is assessed via videoconference and shows her OT how she struggles to get dressed, prepare meals, and set the table at lunch time. Her OT prescribes adaptive clothing and compensatory strategies to help Aysha with her dressing skills. The OT also trains Aysha and her husband on how to use visual cues and environmental modifications to reduce her disability in the kitchen. Aysha’s daughter was also able to join the sessions from a different town and learned strategies to support her mother when visiting on weekends.

## 5. Conclusions

This article summarises current evidence and practice in the delivery of rehabilitation for people with different forms of YOD. A review of the scientific literature shows that there is Class II and Class III evidence that rehabilitative therapies are beneficial for people with YOD. Through examples from high- and low–middle-income countries, we show that the provision of rehabilitation services is possible through a variety of different models and modes of delivery. Setting up rehabilitation services requires a high degree of specialism from clinical leads, both in relation to YOD and nonpharmacological treatments for dementia. This dual expertise is not common but can be built in collaboration with psychologists, OTs, SLTs, and other allied health professionals. An additional common barrier for the provision of YOD rehabilitation therapies across countries and continents is the shortage of resources that impedes Cognitive Disorder and Memory Units to provide services beyond diagnosis and neurology follow-ups. However, as we show in this paper, rehabilitation services can take many forms, be sustainable (e.g., using telehealth), be supported by a wide range of rehabilitation professionals, and can be designed flexibly to adapt to existing resources. We would hope that this paper helps policy makers, commissioners, and clinical leads to see the importance of supporting the development of rehabilitation programmes for people with YOD within existing dementia services.

## Figures and Tables

**Table 1:** Characteristics of four YOD rehabilitation service case studies.

Characteristics	Case Study 1: A Third-Sector Organisation in the UK	Case Study 2: A Private and Public Collaborating Service in Brazil	Case Study 3: A Public Hospital in Chile	Case Study 4: A Public Hospital in India
**Service setting and remit**	The core remit of the charity is to provide long-term support to people with aphasia. The PPA service is a dedicated care pathway within the charity.	The private rehabilitation clinic, SER.COG (non-hospital embedded), sees neurological patients, including those with YOD. The public service is part of a public hospital and public university. For rehabilitation services, only PPA patients are accepted.	The Memory Unit is part of a public hospital. It serves people with dementia, including YOD.	The Cognitive Disorders Clinic sits within the Neurology Department of a public hospital. It serves people with dementia, including YOD.
**Funding system**	Self-funded: users pay a subsidised fee for 1:1 sessions with a specialist SLT and they make a small contribution to peer-support group sessionss (50% reduced fee compared to a private clinic). The charity raises additional funds from grant-making trusts and donations, which enables them to charge less than 50% of the rate of a SLT working in private practice. It also holds a bursary fund which may be used to support people unable to pay.	Private service (SER.COG): Self-funded, with some services provided at a discounted rate. Service costs vary depending on the professional and frequency of sessions, with average monthly costs being USD 250 for OTs and SLTs, and USD 283 for neuropsychology. Public service: publicly funded within the Brazilian Unified Health System.	Funded primarily by the Public Health System, with one administrative staff position funded by the University of Chile’s Faculty of Medicine. The University of Chile also provides the unit’s physical facilities. The service is free for people with dementia with public health insurance living in the area covered by Hospital del Salvador.	Funded by the public health system. Costs are subsidised for people with low socioeconomic status and a Below Poverty Line (BPL) certificate. People with middle to high socioeconomic status pay standard public hospitals charges (in India, the public healthcare system is funded by the government but still requires users to pay for the medical consultation, at a lower rate than in the private sector).
**Referrals and patient flow**	Sixty-five percent of the referrals in 2022-23 were made by family members of people with PPA and the remainder by professionals (SLTs and neurologists). Patient flow: all referrals are screened for suitability by the lead SLT.	Private service (SER.COG) referrals: received from physicians (especially geriatricians, neurologists, and psychiatrist) and self-referrals. Private service (SER.COG) patient flow: After a multidisciplinary assessment, the patient undergoes one to two weekly one-hour rehabilitation sessions until the patient is discharged (often due to disease progression). Patients are managed primarily by the neuropsychologist or OT. The SLT is involved as necessary, with one weekly session. Public service referrals: typically routed through the network from primary and secondary care. Public service—patient flow: availability of rehabilitation is restricted to those diagnosed with PPA, with an average of 12 weekly SLT sessions provided.	The Memory Unit receives referrals from the eight districts of the Eastern Metropolitan Health Service. A referral protocol is established from primary care where YOD patients are consistently referred to the Memory Unit [88]. Patient flow: Once a dementia diagnosis is confirmed, people with YOD are enrolled in a care program that includes interventions aimed at maintaining functionality. The number of sessions is tailored to the specific needs of both the patients and their caregivers [90].	The Cognitive Disorders Clinic receives referrals from neurologists, other medical doctors, and primary health centres across the country and self-referrals. Patient flow: patients with YOD receive diagnostic and multidisciplinary care, including input from neuropsychology, psychiatry, social work, SLTs, OTs, and gait and balance training. The Cognitive Disorders Clinic attends to around 400 people with dementia annually [91].
**Professionals in the service**	Staff: SLT-1 is part-time (Head), and there are two casuals (contribute to group courses and online support as needed). Psychology: links with one independent counselling psychologist (provides individual support and advice on peer group planning).	Private service (SER.COG)—staff: SLT: two; Psychology: one clinical psychologist and one neuropsychologist; OT: two. Private service (SER.COG)—delivery: primarily in-person, with telehealth as an option for those living far from the clinic. Public service—staff: SLT: three; Psychology: seven; OT: three; Physicians: six (neurologists, psychiatrists).	Staff: SLT: one; Psychology: one clinical psychologist and four neuropsychologists; OT: two; Physicians: three neurologists and three psychiatrists; Other: one clinical nurse, one social worker, and two administrative professionals.	Staff: SLT: two; Psychology: two clinical; OT: one; Physicians: three cognitive neurologists; Other: two public health experts, two psychiatric social workers, one geneticists, two imaging specialists, and one physiotherapist.
**Mode of delivery of therapy**	Delivery: mostly in-person; telehealth services available for PPA education and communication partner training for couples and families.	Delivery: services are all in-person.	Delivery: mostly in-person with home visits when necessary. Some interventions (e.g., psychoeducation for caregivers) are available via telehealth.	Delivery: mostly in-person in the Cognitive Neurology ward. Home-based care is provided if required.

YOD: young-onset dementia; PPA: primary progressive aphasia; SLT: speech and language therapist; OT: occupational therapist; UFMG: Universidade Federal de Minas Gerais.

## Data Availability

The original contributions presented in the study are included in the article, further inquiries can be directed to the corresponding author.

## References

[1] WHO Rehabilitation. https://www.who.int/news-room/fact-sheets/detail/rehabilitation.

[2] The Lancet Neurology (2023). Brain health, disability, and the need for broader thinking. Lancet Neurol..

[3] Kudlicka A., Martyr A., Bahar-Fuchs A., Sabates J., Woods B., Clare L. (2023). Cognitive rehabilitation for people with mild to moderate dementia. Cochrane Database Syst. Rev..

[4] Jeon Y.-H., Simpson J.M., Low L.-F., Woods R., Norman R., Mowszowski L., Clemson L., Naismith S.L., Brodaty H., Hilmer S. (2019). A pragmatic randomised controlled trial (RCT) and realist evaluation of the interdisciplinary home-bAsed Reablement program (I-HARP) for improving functional independence of community dwelling older people with dementia: An effectiveness-implementation hybrid design. BMC Geriatr..

[5] Clemson L., Laver K., Rahja M., Culph J., Scanlan J.N., Day S., Comans T., Jeon Y.-H., Low L.-F., Crotty M. (2021). Implementing a Reablement Intervention, “Care of People with Dementia in Their Environments (COPE)”: A Hybrid Implementation-Effectiveness Study. Gerontol..

[6] Gitlin L.N., Corcoran M., Winter L., Boyce A., Hauck W.W. (2001). A Randomized, Controlled Trial of a Home Environmental Intervention. Gerontol..

[7] WHO Package of Interventions for Rehabilitation: Module 3: Neurological Conditions. https://www.who.int/publications/i/item/9789240071131.

[8] Poulos C.J., Bayer A., Beaupre L., Clare L., Poulos R.G., Wang R.H., Zuidema S., McGilton K.S. (2017). A comprehensive approach to reablement in dementia. Alzheimer’s Dement. Transl. Res. Clin. Interv..

[9] Clare L. (2017). Rehabilitation for people living with dementia: A practical framework of positive support. PLoS Med..

[10] Jogie P., Rahja M., Berg M.v.D., Cations M., Brown S., Laver K. (2021). Goal setting for people with mild cognitive impairment or dementia in rehabilitation: A scoping review. Aust. Occup. Ther. J..

[11] O’Connor C.M.C., Rowlands A., Poulos C.J. (2022). Development of an assessment guide to evaluate meaningful outcomes for people living with dementia who are engaged in reablement programs. Disabil. Rehabil..

[12] Rossor M.N., Fox N.C., Mummery C.J., Schott J.M., Warren J.D. (2010). The diagnosis of young-onset dementia. Lancet Neurol..

[13] Hendriks S., Peetoom K., Bakker C., van der Flier W.M., Papma J.M., Koopmans R., Verhey F.R.J., de Vugt M., Köhler S., Withall A. (2021). Global Prevalence of Young-Onset Dementia. JAMA Neurol..

[14] Kilty C., Cahill S., Foley T., Fox S. (2022). Young onset dementia: Implications for employment and finances. Dementia.

[15] Mayrhofer A.M., Greenwood N., Smeeton N., Almack K., Buckingham L., Shora S., Goodman C. (2021). Understanding the financial impact of a diagnosis of young onset dementia on individuals and families in the United Kingdom: Results of an online survey. Health Soc. Care Community.

[16] McKhann G.M., Knopman D.S., Chertkow H., Hyman B.T., Jack C.R., Kawas C.H., Klunk W.E., Koroshetz W.J., Manly J.J., Mayeux R. (2011). The diagnosis of dementia due to Alzheimer’s disease: Recommendations from the National Institute on Aging-Alzheimer’s association workgroups on diagnostic guidelines for Alzheimer’s disease. Alzheimers Dement. J. Alzheimers Assoc..

[17] Dubois B., Villain N., Frisoni G.B., Rabinovici G.D., Sabbagh M., Cappa S., Bejanin A., Bombois S., Epelbaum S., Teichmann M. (2021). Clinical diagnosis of Alzheimer’s disease: Recommendations of the International Working Group. Lancet Neurol..

[18] Rascovsky K., Hodges J.R., Knopman D., Mendez M.F., Kramer J.H., Neuhaus J., van Swieten J.C., Seelaar H., Dopper E.G., Onyike C.U. (2011). Sensitivity of revised diagnostic criteria for the behavioural variant of frontotemporal dementia. Brain.

[19] Gorno-Tempini M.L., Hillis A.E., Weintraub S., Kertesz A., Mendez M., Cappa S.F., Ogar J.M., Rohrer J.D., Black S., Boeve B.F. (2011). Classification of primary progressive aphasia and its variants. Neurology.

[20] Tang-Wai D.F., Graff-Radford N.R., Boeve B.F., Dickson D.W., Parisi J.E., Crook R., Caselli R.J., Knopman D.S., Petersen R.C. (2004). Clinical, genetic, and neuropathologic characteristics of posterior cortical atrophy. Neurology.

[21] Crutch S.J., Schott J.M., Rabinovici G.D., Murray M., Snowden J.S., van der Flier W.M., Dickerson B.C., Vandenberghe R., Ahmed S., Bak T.H. (2017). Consensus classification of posterior cortical atrophy. Alzheimer’s Dement..

[22] Hammers D.B., Eloyan A., Taurone A., Thangarajah M., Beckett L., Gao S., Kirby K., Aisen P., Dage J.L., Foroud T. (2023). Profiling baseline performance on the Longitudinal Early-Onset Alzheimer’s Disease Study (LEADS) cohort near the midpoint of data collection. Alzheimer’s Dement..

[23] Aplaon M., Belchior P., Gélinas I., Bier N., Aboujaoudé A. (2016). Interventions for Individuals with Young-Onset Dementia. A Review of the Literature. J. Aging Res. Clin. Pract..

[24] Kim I., Yang Y., Cheon H., Kim J., Song J. (2024). Non-pharmacological interventions for people living with young-onset dementia and their carers: A scoping review focussing on the support of participants’ needs. J. Psychiatr. Ment. Health Nurs..

[25] Mayrhofer A., Mathie E., McKeown J., Bunn F., Goodman C. (2018). Age-appropriate services for people diagnosed with young onset dementia (YOD): A systematic review. Aging Ment. Health.

[26] Clare L., Linden D.E., Woods R.T., Whitaker R., Evans S.J., Parkinson C.H., van Paasschen J., Nelis S.M., Hoare Z., Yuen K.S. (2010). Goal-Oriented Cognitive Rehabilitation for People with Early-Stage Alzheimer Disease: A Single-Blind Randomized Controlled Trial of Clinical Efficacy. Am. J. Geriatr. Psychiatry.

[27] Clare L., Kudlicka A., Oyebode J.R., Jones R.W., Bayer A., Leroi I., Kopelman M., James I.A., Culverwell A., Pool J. (2019). Individual goal-oriented cognitive rehabilitation to improve everyday functioning for people with early-stage dementia: A multicentre randomised controlled trial (the GREAT trial). Int. J. Geriatr. Psychiatry.

[28] Clare L., Kudlicka A., Oyebode J.R., Jones R.W., Bayer A., Leroi I., Kopelman M., James I.A., Culverwell A., Pool J. (2019). Goal-oriented cognitive rehabilitation for early-stage Alzheimer’s and related dementias: The GREAT RCT. Health Technol. Assess..

[29] Bahar-Fuchs A., Clare L., Woods B. (2013). Cognitive training and cognitive rehabilitation for mild to moderate Alzheimer’s disease and vascular dementia. Cochrane Database Syst. Rev..

[30] Hofmann M., Hock C., Kühler A., Müller-Spahn F. (1996). Interactive computer-based cognitive training in patients with Alzheimer’s disease. J. Psychiatr. Res..

[31] Tonga J.B., Karlsoeen B.B., Arnevik E.A., Werheid K., Korsnes M.S., Ulstein I.D. (2016). Challenges With Manual-Based Multimodal Psychotherapy for People With Alzheimer’s Disease. Am. J. Alzheimer’s Dis. Other Dementiasr..

[32] Arntzen C., Holthe T., Jentoft R. (2016). Tracing the successful incorporation of assistive technology into everyday life for younger people with dementia and family carers. Dementia.

[33] Holthe T., Halvorsrud L., Karterud D., Hoel K.-A., Lund A. (2018). Usability and acceptability of technology for community-dwelling older adults with mild cognitive impairment and dementia: A systematic literature review. Clin. Interv. Aging.

[34] Robertson J., Evans D. (2015). Evaluation of a workplace engagement project for people with younger onset dementia. J. Clin. Nurs..

[35] Robertson J., Evans D., Horsnell T. (2013). Side by Side: A workplace engagement program for people with younger onset dementia. Dementia.

[36] Salech G.M., Lillo P., van der Hiele K., Méndez-Orellana C., Ibáñez A., Slachevsky A. (2022). Apathy, Executive Function, and Emotion Recognition Are the Main Drivers of Functional Impairment in Behavioral Variant of Frontotemporal Dementia. Front. Neurol..

[37] Barton C., Ketelle R., Merrilees J., Miller B. (2016). Non-pharmacological Management of Behavioral Symptoms in Frontotemporal and Other Dementias. Curr. Neurol. Neurosci. Rep..

[38] Lough S., Hodges J.R. (2002). Measuring and modifying abnormal social cognition in frontal variant frontotemporal dementia. J. Psychosom. Res..

[39] Tanabe H., Ikeda M., Komori K. (1999). Behavioral Symptomatology and Care of Patients with Frontotemporal Lobe Degeneration—Based on the Aspects of the Phylogenetic and Ontogenetic Processes. Dement. Geriatr. Cogn. Disord..

[40] Lima-Silva T.B., Bahia V.S., Nitrini R., Yassuda M.S. (2013). Functional Status in Behavioral Variant Frontotemporal Dementia: A Systematic Review. BioMed. Res. Int..

[41] O’Connor C.M., Clemson L., da Silva T.B.L., Piguet O., Hodges J.R., Mioshi E. (2013). Enhancement of carer skills and patient function in the non-pharmacological management of frontotemporal dementia (FTD): A call for randomised controlled studies. Dement. Neuropsychol..

[42] O’Connor C.M., Clemson L., Brodaty H., Low L.-F., Jeon Y.-H., Gitlin L.N., Piguet O., Mioshi E. (2019). The tailored activity program (TAP) to address behavioral disturbances in frontotemporal dementia: A feasibility and pilot study. Disabil. Rehabil..

[43] Vlotinou P., Tsiakiri A., Detsaridou G., Nikova A., Tsiptsios D., Vadikolias K., Aggelousis N. (2023). Occupational Therapy Interventions in Patients with Frontotemporal Dementia: A Systematic Review. Med. Sci..

[44] Gitlin L.N., Winter L., Earland T.V., Herge E.A., Chernett N.L., Piersol C.V., Burke J.P. (2009). The Tailored Activity Program to Reduce Behavioral Symptoms in Individuals with Dementia: Feasibility, Acceptability, and Replication Potential. The Gerontologist.

[45] O’Connor C.M., Clemson L., Brodaty H., Gitlin L.N., Piguet O., Mioshi E. (2016). Enhancing caregivers’ understanding of dementia and tailoring activities in frontotemporal dementia: Two case studies. Disabil. Rehabil..

[46] Godefroy V., Batrancourt B., Charron S., Bouzigues A., Sezer I., Bendetowicz D., Carle G., Rametti-Lacroux A., Bombois S., Cognat E. (2022). Disentangling Clinical Profiles of Apathy in Behavioral Variant Frontotemporal Dementia. J. Alzheimer’s Dis..

[47] O’Connor C.M., Landin-Romero R., Clemson L., Kaizik C., Daveson N., Hodges J.R., Hsieh S., Piguet O., Mioshi E. (2017). Behavioral-variant frontotemporal dementia. Neurology.

[48] Fisher A.C., Cheung S.C., O’Connor C.M.C., Piguet O. (2023). The Acceptability and Usefulness of Positive Behaviour Support Education for Family Carers of People with Frontotemporal Dementia: A Pilot Study. J. Geriatr. Psychiatry Neurol..

[49] O’Connor C.M.C., Mioshi E., Kaizik C., Fisher A., Hornberger M., Piguet O. (2021). Positive behaviour support in frontotemporal dementia: A pilot study. Neuropsychol. Rehabil..

[50] Ruggero L., Nickels L., Croot K. (2019). Quality of life in primary progressive aphasia: What do we know and what can we do next?. Aphasiology.

[51] O’Connor C.M., Ahmed S., Mioshi E. (2014). Functional disability in primary progressive aphasia. Aphasiology.

[52] Butts A.M., Machulda M.M., Duffy J.R., Strand E.A., Whitwell J.L., Josephs K.A. (2015). Neuropsychological Profiles Differ among the Three Variants of Primary Progressive Aphasia. J. Int. Neuropsychol. Soc..

[53] de la Sablonnière J., Tastevin M., Lavoie M., Laforce R. (2021). Longitudinal Changes in Cognition, Behaviours, and Functional Abilities in the Three Main Variants of Primary Progressive Aphasia: A Literature Review. Brain Sci..

[54] Cotelli M., Manenti R., Ferrari C., Gobbi E., Macis A., Cappa S.F. (2020). Effectiveness of language training and non-invasive brain stimulation on oral and written naming performance in Primary Progressive Aphasia: A meta-analysis and systematic review. Neurosci. Biobehav. Rev..

[55] Wauters L.D., Croot K., Dial H.R., Duffy J.R., Grasso S.M., Kim E., Mendez K.S., Ballard K.J., Clark H.M., Kohley L. (2023). Behavioral Treatment for Speech and Language in Primary Progressive Aphasia and Primary Progressive Apraxia of Speech: A Systematic Review. Neuropsychol. Rev..

[56] Suárez-González A., Savage S.A., Bier N., Henry M.L., Jokel R., Nickels L., Taylor-Rubin C. (2021). Semantic Variant Primary Progressive Aphasia: Practical Recommendations for Treatment from 20 Years of Behavioural Research. Brain Sci..

[57] Taylor-Rubin C., Azizi L., Croot K., Nickels L. (2020). Primary Progressive Aphasia Education and Support Groups: A Clinical Evaluation. Am. J. Alzheimer’s Dis. Other Dementiasr..

[58] Taylor-Rubin C., Croot K., Nickels L. (2019). Adherence to lexical retrieval treatment in Primary Progressive Aphasia and implications for candidacy. Aphasiology.

[59] Savage S.A., Piguet O., Hodges J.R. (2014). Giving words new life: Generalization of word retraining outcomes in semantic dementia. J. Alzheimer’s Dis..

[60] Savage S.A.M., Piguet O., Hodges J.R.F. (2015). Cognitive Intervention in Semantic Dementia. Alzheimer Dis. Assoc. Disord..

[61] Machado T.H., Carthery-Goulart M.T., Campanha A.C., Caramelli P. (2021). Cognitive Intervention Strategies Directed to Speech and Language Deficits in Primary Progressive Aphasia: Practice-Based Evidence from 18 Cases. Brain Sci..

[62] Grasso S.M., Rodríguez C.A.W., Colomer N.M., Kiderle S.-K.M., Sánchez-Valle R., Santos M.S. (2023). Bilingual Primary Progressive Aphasia: A Scoping Review of Assessment and Treatment Practices. J. Alzheimer’s Dis..

[63] Henry M.L., Hubbard H.I., Grasso S.M., Mandelli M.L., Wilson S.M., Sathishkumar M.T., Fridriksson J., Daigle W., Boxer A.L., Miller B.L. (2018). Retraining speech production and fluency in non-fluent/agrammatic primary progressive aphasia. Brain.

[64] Lavoie M., Bier N., Laforce R., Macoir J. (2020). Improvement in functional vocabulary and generalization to conversation following a self-administered treatment using a smart tablet in primary progressive aphasia. Neuropsychol. Rehabil..

[65] Meyer A.M., Getz H.R., Brennan D.M., Hu T.M., Friedman R.B. (2016). Telerehabilitation of anomia in primary progressive aphasia. Aphasiology.

[66] Meyer A.M., Tippett D.C., Friedman R.B. (2018). Prophylaxis and remediation of anomia in the semantic and logopenic variants of primary progressive aphasia. Neuropsychol. Rehabil..

[67] Meyer A.M., Snider S.F., Eckmann C.B., Friedman R.B. (2015). Prophylactic treatments for anomia in the logopenic variant of primary progressive aphasia: Cross-language transfer. Aphasiology.

[68] Volkmer A., Spector A., Warren J.D., Beeke S. (2019). Speech and language therapy for primary progressive aphasia across the UK: A survey of current practice. Int. J. Lang. Commun. Disord..

[69] Folder N., Power E., Rietdijk R., Christensen I., Togher L., Parker D. (2024). The Effectiveness and Characteristics of Communication Partner Training Programs for Families of People with Dementia: A Systematic Review. The Gerontologist.

[70] Jokel R., Meltzer J. (2017). Group intervention for individuals with primary progressive aphasia and their spouses: Who comes first?. J. Commun. Disord..

[71] Beale N., Fried-Oken M., Mooney A. (2018). Group Communication Treatment for Individuals with PPA and Their Partners. Semin. Speech Lang..

[72] Morhardt D.J., O’hara M.C., Zachrich K., Wieneke C., Rogalski E.J. (2019). Development of a Psycho-Educational Support Program for Individuals with Primary Progressive Aphasia and their Care-Partners. Dementia.

[73] Schaffer K.M., Henry M.L. (2023). Implementing a telehealth-delivered psychoeducational support group for care partners of individuals with primary progressive aphasia. Aphasiology.

[74] Shakespeare T.J., Yong K.X., Foxe D., Hodges J., Crutch S.J. (2014). Pronounced Impairment of Everyday Skills and Self-Care in Posterior Cortical Atrophy. J. Alzheimer’s Dis..

[75] Ahmed S., Culley S., Blanco-Duque C., Hodges J.R., Butler C., Mioshi E. (2020). Pronounced Impairment of Activities of Daily Living in Posterior Cortical Atrophy. Dement. Geriatr. Cogn. Disord..

[76] Crutch S.J., Warrington E.K. (2007). Foveal crowding in posterior cortical atrophy: A specific early-visual-processing deficit affecting word reading. Cogn. Neuropsychol..

[77] Yong K.X., Rajdev K., Shakespeare T.J., Leff A.P., Crutch S.J. (2015). Facilitating text reading in posterior cortical atrophy. Neurology.

[78] Bier N., El-Samra A., Bottari C., Vallet G., Carignan M., Paquette G., Brambati S., Demers L., Génier-Marchand D., Rouleau I. (2019). Posterior cortical atrophy: Impact on daily living activities and exploration of a cognitive rehabilitation approach. Cogent Psychol..

[79] Roca M., Gleichgerrcht E., Torralva T., Manes F. (2010). Cognitive rehabilitation in posterior cortical atrophy. Neuropsychol. Rehabil..

[80] Weill-Chounlamountry A., Alves J., Pradat-Diehl P. (2016). Non-pharmacological intervention for posterior cortical atrophy. World J. Clin. Cases.

[81] Suarez-Gonzalez A., Ocal D., Pavisic I., Peacock A., Naessens M., Ahmed S., Butler C.R., Leff A.P., Yong K.X., Crutch S.J. (2019). ReadClear: An Assistive Reading Tool for People Living with Posterior Cortical Atrophy. J. Alzheimer’s Dis..

[82] Scheiman M., Scheiman M., Whittaker S. (2015). Low Vision Rehabilitation: A Practical Guide for Occupational Therapists.

[83] Yong K.X.X., Graff-Radford J., Ahmed S., Chapleau M., Ossenkoppele R., Putcha D., Rabinovici G.D., Suarez-Gonzalez A., Schott J.M., Crutch S. (2023). Diagnosis and Management of Posterior Cortical Atrophy. Curr. Treat. Options Neurol..

[84] Yong K.X.X., McCarthy I.D., Poole T., Suzuki T., Yang B., Carton A.M., Holloway C., Papadosifos N., Boampong D., Langham J. (2018). Navigational cue effects in Alzheimer’s disease and posterior cortical atrophy. Ann. Clin. Transl. Neurol..

[85] Sau-Fun N., Chi-Leung H., Lai-Fan W. (2011). Development of medical garments and apparel for the elderly and the disabled. Text. Prog..

[86] Beresford B.A., Mann R.C., Parker G.M., Kanaan M., Neves De Faria R.I., Rabiee P., Weatherly H., Clarke S., Mayhew E., Duarte A. (2019). Bryony Beresford, Reablement Services for People at Risk of Needing Social Care: The MoRe Mixed-Methods Evaluation.

[87] London Clinical Network Young Onset Dementia; Examples of Post Diagnostic Support Across London. https://www.england.nhs.uk/london/wp-content/uploads/sites/8/2019/10/YOD-examples-of-post-diagnostic-support-London.pdf.

[88] Leon T., Castro L., Mascayano F., Lawlor B., Slachevsky A. (2021). Evaluating a Memory Clinic Using the RE-AIM Model. The Experience of the “Memory and Neuropsychiatry Clinic” in Hospital Del Salvador, Chile. Front. Neurol..

[89] Stamou V., La Fontaine J., O’Malley M., Jones B., Parkes J., Carter J., Oyebode J.R. (2022). Helpful post-diagnostic services for young onset dementia: Findings and recommendations from the Angela project. Health Soc. Care Community.

[90] Slachevsky Chonchol A., Ramos Franco T. (2023). Guía de funcionamiento Unidad de Memoria: Centro de Memoria y Neuropsiquiatría.

[91] Arshad F., Alladi S. (2024). The Most Difficult Question in a Cognitive Disorders Clinic. JAMA Neurol..

[92] Chrysanthaki T., Fernandes B., Smith S., Black N. (2017). Can Memory Assessment Services (MAS) in England be categorized? A national survey. J. Public Health.

[93] Doncaster E., McGeorge M., Orrell M. (2011). Developing and implementing quality standards for memory services: The Memory Services National Accreditation Programme (MSNAP). Aging Ment. Health.

[94] Cook L., Souris H., Isaacs J. (2020). The 2019 National Memory Service Audit.

[95] Khayum B., Rogalski E.J. (2018). A Life Participation Approach to Primary Progressive Aphasia Intervention. Semin. Speech Lang..

[96] Thumala D., Kennedy B.K., Calvo E., Gonzalez-Billault C., Zitko P., Lillo P., Villagra R., Ibáñez A., Assar R., Andrade M. (2017). Aging and Health Policies in Chile: New Agendas for Research. Health Syst. Reform.

[97] Custodio N., Wheelock A., Thumala D., Slachevsky A. (2017). Dementia in Latin America: Epidemiological Evidence and Implications for Public Policy. Front. Aging Neurosci..

[98] Chokshi M., Patil B., Khanna R., Neogi S.B., Sharma J., Paul V.K., Zodpey S. (2016). Health systems in India. J. Perinatol..

[99] Goyal S., Darshini K., Arshad F., Varghese F.A., Paplikar A., Vandana V., Ramakrishnan S., Alladi S. (2021). Language profile of primary progressive aphasia in a linguistically diverse cohort in India. Alzheimer’s Dement..

[100] WHO (2017). Global Diffusion of eHealth: Making Universal Health Coverage Achievable: Report of the Third Global Survey on eHealth.

[101] Brown A.D., Kelso W., Velakoulis D., Farrand S., Stolwyk R.J. (2023). Understanding Clinician’s Experiences with Implementation of a Younger Onset Dementia Telehealth Service. J. Geriatr. Psychiatry Neurol..

[102] O’connell M.E., Crossley M., Cammer A., Morgan D., Allingham W., Cheavins B., Dalziel D., Lemire M., Mitchell S., Morgan E. (2014). Development and evaluation of a telehealth videoconferenced support group for rural spouses of individuals diagnosed with atypical early-onset dementias. Dementia.

[103] Perin S., Lai R., Diehl-Schmid J., You E., Kurz A., Tensil M., Wenz M., Foertsch B., Lautenschlager N.T. (2023). Online counselling for family carers of people with young onset dementia: The RHAPSODY-Plus pilot study. Digit. Health.

[104] Rao L.A., Roberts A.C., Schafer R., Rademaker A., Blaze E., Esparza M., Salley E., Coventry C., Weintraub S., Mesulam M.-M. (2022). The Reliability of Telepractice Administration of the Western Aphasia Battery–Revised in Persons with Primary Progressive Aphasia. Am. J. Speech-Lang. Pathol..

[105] Roberts A.C., Rademaker A.W., Salley E.A., Mooney A., Morhardt D., Fried-Oken M., Weintraub S., Mesulam M., Rogalski E. (2022). Communication Bridge™-2 (CB2): An NIH Stage 2 randomized control trial of a speech-language intervention for communication impairments in individuals with mild to moderate primary progressive aphasia. Trials.

[106] Wong B., Loyer E., Sullivan C., Krivensky S., Lopez A.V., Quimby M., Hochberg D., Dev S.I., Putcha D., Eldaief M.C. (2021). Feasibility of multidisciplinary telehealth evaluations in atypical dementia. Alzheimer’s Dement..

[107] Cotelli M., Manenti R., Brambilla M., Gobbi E., Ferrari C., Binetti G., Cappa S.F. (2019). Cognitive telerehabilitation in mild cognitive impairment, Alzheimer’s disease and frontotemporal dementia: A systematic review. J. Telemed. Telecare.

[108] Rhodus E.K., Baum C., Kryscio R., Liu C., George R., Thompson M., Lowry K., Coy B., Barber J., Nichols H. (2023). Feasibility of Telehealth Occupational Therapy for Behavioral Symptoms of Adults with Dementia: Randomized Controlled Trial. Am. J. Occup. Ther..

[109] Rogalski E.J., Saxon M., McKenna H., Wieneke C., Rademaker A., Corden M.E., Borio K., Mesulam M., Khayum B. (2016). Communication Bridge: A pilot feasibility study of Internet-based speech–language therapy for individuals with progressive aphasia. Alzheimer’s Dement. Transl. Res. Clin. Interv..

[110] Beeke S., Volkmer A., Farrington-Douglas C. (2021). TeleCPT: Delivery of a Better Conversations Approach to Communication Partner Training During a Global Pandemic and Beyond. Perspect. ASHA Spéc. Interes. Groups.

[111] Dial H.R., Hinshelwood H., Grasso S.M., Hubbard H.I., Gorno-Tempini M.-L., Henry M.L. (2019). Investigating the utility of teletherapy in individuals with primary progressive aphasia. Clin. Interv. Aging.

[112] Clare L., Kudlicka A., Collins R., Evans S., Pool J., Henderson C., Knapp M., Litherland R., Oyebode J., Woods R. (2023). Implementing a home-based personalised cognitive rehabilitation intervention for people with mild-to-moderate dementia: GREAT into Practice. BMC Geriatr..

[113] ITU Measuring Digital Development: Facts and Figures 2023. https://www.itu.int/en/ITU-D/Statistics/Pages/facts/default.aspx.

[114] Wilson J., Heinsch M., Betts D., Booth D., Kay-Lambkin F. (2021). Barriers and facilitators to the use of e-health by older adults: A scoping review. BMC Public Health.

[115] Mamedova S., Pawlowski E., Hudson L. (2018). A Description of US Adults Who Are Not Digitally Literate.

